# Total Laparoscopic Hysterectomy in Factor XI Deficiency: A Case Report

**DOI:** 10.7759/cureus.97930

**Published:** 2025-11-27

**Authors:** Brayan J Ortiz-Villanueva, Alejandra G Lopez-Chavez, Alejandra Mendoza-Torres, Luz M Bravo-Rodriguez, Fernando J Interián-Alvarez

**Affiliations:** 1 Gynecological Endoscopic Surgery, Hospital Regional Adolfo López Mateos, Institute for Social Security and Services for State Workers, Mexico City, MEX; 2 Obstetrics and Gynaecology, La Salle University, Mexico City, MEX; 3 Department of Hematology, Hospital Regional Adolfo López Mateos, Institute for Social Security and Services for State Workers, Mexico City, MEX; 4 Obstetrics and Gynaecology, Hospital Regional Elvia Carrillo Puerto, Institute for SocialSecurity and Services for State Workers, Yucatan, MEX

**Keywords:** abnormal uterine bleeding, coagulopathy, factor xi deficiency, haemophilia c, total laparoscopic hysterectomy

## Abstract

Abnormal uterine bleeding (AUB) is a frequent reason for gynaecological consultation and can occasionally reflect rare coagulation disorders. The case study presented herein concerns a 42-year-old para 3 woman (gravida 3, para 3), who exhibited symptoms including irregular heavy menses and the presence of a large uterine myoma. The patient was referred for further evaluation due to symptomatic anaemia and a prolonged activated partial thromboplastin time (aPTT). Haematological evaluation confirmed severe factor XI (FXI) deficiency, with activity levels of 0.3% and 1.24% on repeat testing.

As medical therapy proved ineffective in controlling the bleeding over several months, a total laparoscopic hysterectomy (TLH) was planned to minimise the risk of haemorrhage. Preoperative management included the intravenous administration of six units of fresh frozen plasma (FFP) over 24 hours, together with tranexamic acid. The procedure was successfully completed with minimal blood loss (approximately 80 mL), and the patient was discharged 48 hours postoperatively without complications.

This case demonstrates the significance of identifying inherited coagulopathies as underlying causes of AUB and underscores the pivotal role of multidisciplinary collaboration, meticulous perioperative planning, and minimally invasive surgical techniques in optimising outcomes and mitigating bleeding risk in women with FXI deficiency.

## Introduction

Abnormal uterine bleeding (AUB) is a common reason for gynaecological consultation and can significantly impair women’s quality of life. Although hormonal and structural abnormalities are usually the predominant causes, disorders of haemostasis must also be considered as potential underlying aetiologies. Among these, factor XI (FXI) deficiency-also known as haemophilia C-represents a rare but clinically relevant cause of excessive gynaecological bleeding [1-3].

Congenital FXI deficiency affects approximately one person per million in the general population, making it an uncommon coagulopathy [4]. However, its prevalence is considerably higher in specific populations, such as Ashkenazi Jews, where up to one in 450 individuals present with severe forms of the disorder, and between 5 % and 10 % are heterozygous carriers [3,5].

FXI is a plasma protein that plays a key role in amplifying the intrinsic coagulation cascade, and its deficiency can result in variable haemorrhagic manifestations [3,6]. Unlike other hereditary bleeding disorders such as haemophilia A or B, FXI deficiency exhibits an unpredictable relationship between plasma factor levels and the clinical severity of bleeding [5,6]. In affected women, it may manifest as alterations in menstrual volume, duration, and frequency, intermenstrual bleeding, postpartum haemorrhage, or bleeding complications during gynaecological and obstetric procedures-highlighting the importance of early recognition and multidisciplinary management [2,3].

The use of surgical techniques that enhance safety and minimise bleeding risk is also recommended for these patients. With the advent of minimally invasive surgery, the therapeutic landscape of gynaecology has been transformed: laparoscopic procedures provide multiple advantages over laparotomy, including reduced intraoperative blood loss, less postoperative pain, faster recovery, and shorter hospitalisation. These benefits are particularly valuable in patients with coagulopathies [7,8].

Despite its clinical significance, the literature on the impact of FXI deficiency within the gynaecological-obstetric context remains limited, particularly in Latin American populations. This article analyses the clinical presentation, diagnostic approach, and therapeutic strategies in women with AUB associated with FXI deficiency, aiming to enhance recognition of this rare entity, improve outcomes, and reduce morbidity among affected patients.

## Case presentation

A 42-year-old woman, blood type A Rh-positive, para 3 (gravida 3, para 3), with a history of three caesarean deliveries due to cephalopelvic disproportion, last complicated by obstetric haemorrhage evaluated for abnormal uterine bleeding (AUB). She was not pregnant at the time of evaluation. Her medical history included a penicillin allergy, severe anaemia previously treated with intravenous iron dextran and oral haematinics, and untreated Eustachian tube dysfunction.

Six months before admission, she noted changes in her menstrual pattern characterised by heavy bleeding every 28 days, lasting five days, requiring up to six large sanitary pads daily (estimated volume ≈ 600 mL), associated with large clots and incapacitating dysmenorrhoea refractory to analgesics.

She was referred to a tertiary-care centre with a diagnosis of AUB secondary to uterine fibroids and symptomatic anaemia. On admission, she presented with symptoms of low cardiac output and anaemia syndrome. Laboratory tests revealed severe microcytic hypochromic anaemia and prolonged activated partial thromboplastin time (aPTT). She received two units of packed red blood cells and one unit of fresh frozen plasma (FFP). A haematology consultation was requested for multidisciplinary management.

After transfusion, her haemoglobin rose to 9.4 g/dL, yet the aPTT remained prolonged at 45s. Correction testing yielded a Rosner index of 2, suggestive of a coagulation-factor deficiency. A comprehensive coagulopathy panel was initiated to evaluate factors VIII, IX, and von Willebrand factor (vWF).

**Figure 1:**
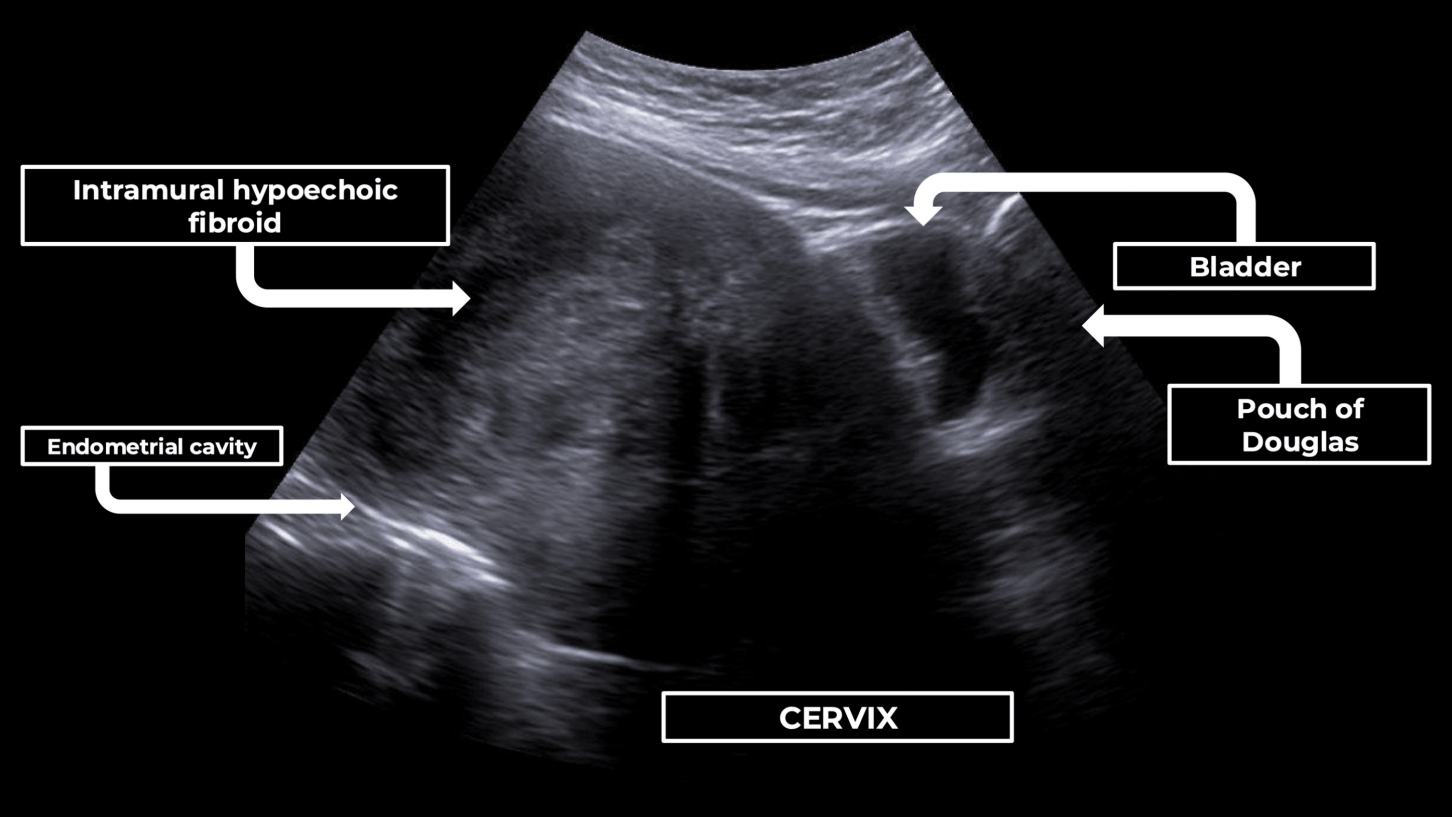
Pelvic ultrasound Showing a hypoechoic intramural uterine fibroid (FIGO type 4–5) and normal anatomic orientation of the bladder, cervix, and pouch of Douglas. According to the FIGO classification system (PALM-COEIN) for abnormal uterine bleeding (Munro et al., 2011 [9]). FIGO: International Federation of Gynecology and Obstetrics; PALM-COEIN: Polyp, Adenomyosis, Leiomyoma, Malignancy, Coagulopathy, Ovulatory Dysfunction, Endometrial, Iatrogenic, and Not-yet-classified

The patient’s anaemia improved with intravenous iron infusions. Pelvic ultrasonography (Figure 1) revealed an anteverted uterus measuring 11.8 × 8.1 × 10.3 cm, containing a dominant ovoid hypoechoic lesion measuring 9.2 × 7.2 × 7.1 cm in the uterine body and fundus (FIGO 4-5), and an adjacent smaller lesion measuring 3.4 × 1.8 cm (FIGO 2-3) [9]. The arrows in Figure 1 have been refined by reducing their thickness to improve visual clarity without compromising anatomical identification.

**Figure 2:**
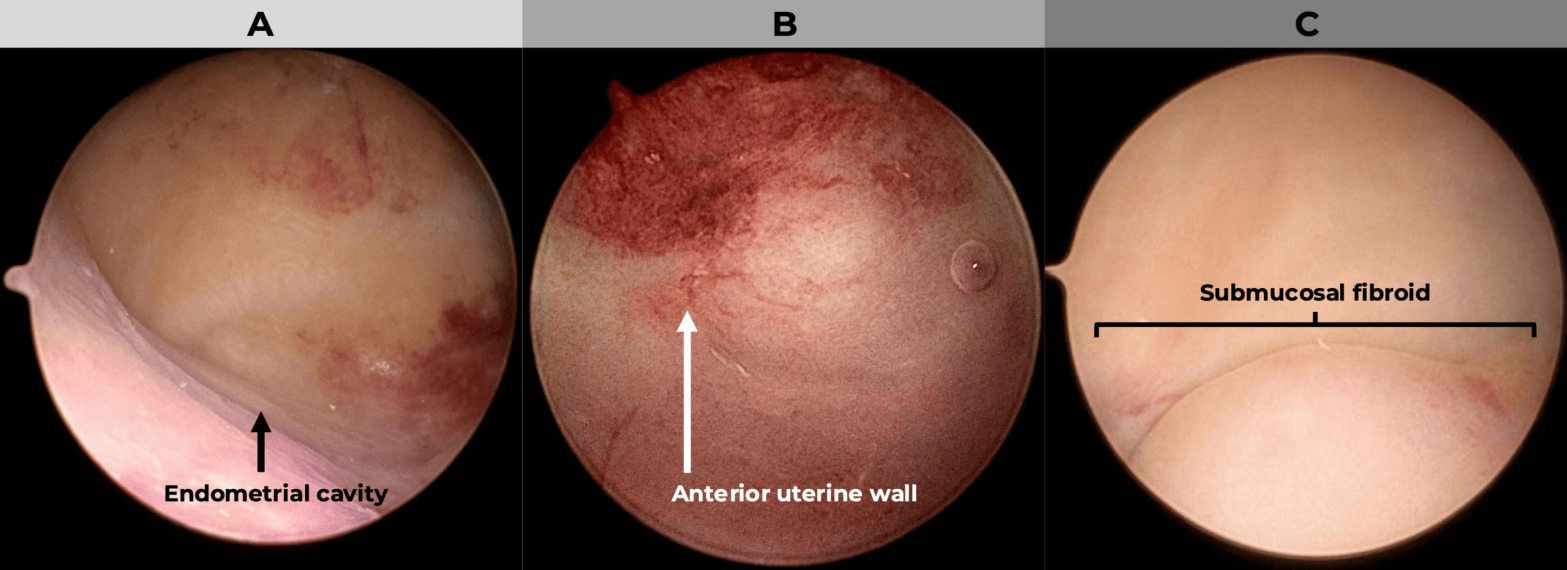
Sequential hysteroscopic Sequential hysteroscopic views showing (A) the endometrial cavity with normal mucosal appearance; (B) the anterior uterine wall; and (C) a submucosal fibroid (FIGO type 2) protruding into the uterine lumen. According to the FIGO classification system (PALM-COEIN) for abnormal uterine bleeding (Munro et al., 2011 [9]). FIGO: International Federation of Gynecology and Obstetrics; PALM-COEIN: Polyp, Adenomyosis, Leiomyoma, Malignancy, Coagulopathy, Ovulatory Dysfunction, Endometrial, Iatrogenic, and Not-yet-classified

An office hysteroscopy demonstrated a markedly enlarged uterine cavity with submucosal fibroid impressions (FIGO 2), the largest approximately 2 cm. Endometrial biopsy showed inactive endometrium and unremarkable myometrium (Figure 2) [9].

Haematologic testing revealed reduced platelet aggregation with epinephrine and arachidonic acid, and a transient decrease in factor VIII activity. A therapeutic trial with recombinant factor VIII produced no improvement in uterine bleeding. Screening for factor VIII inhibitors was negative on two occasions, and assays for vWF antigen, ristocetin cofactor, and fibrinogen were within normal limits.

Measurement of factor XI activity revealed a severe deficiency (0.3%), confirmed by repeat testing (1.24%), establishing the diagnosis of congenital factor XI deficiency. Both measurements were performed in the same laboratory using the same assay method and reference range; the slight variation is attributable to expected inter-assay variability and the timing of sampling following plasma transfusion. Importantly, both values fall within the range of severe FXI deficiency and do not alter the clinical interpretation.

Given persistent bleeding despite medical therapy and the increased haemorrhagic risk, a total laparoscopic hysterectomy (TLH) with bilateral salpingectomy was planned to achieve definitive control. Preoperative management included tranexamic acid 1 g every 8h and FFP (two units every 8h), initiated 24h before surgery.

Intraoperatively, the uterus measured approximately 26×20×12 cm, globular in shape, with normal adnexa. Dense bladder adhesions to the uterine isthmus and fibrosis along the uterosacral ligaments were noted. Intraoperative blood loss was minimal (≈ 80 mL), and a pelvic drain was placed.

Postoperatively, the patient remained stable. The haematology team recommended continuing tranexamic acid 1 g every 8 h for three doses and administering two additional FFP units to reduce the risk of delayed bleeding. She was discharged 48 hours after surgery without postoperative complications (Table 1).

**Table 1:** Laboratory findings in this patient and reference ranges. Laboratory findings demonstrating prolonged aPTT and severe factor XI deficiency with preserved platelet count, vWF and FVIII within acceptable ranges. Values support a diagnosis of congenital FXI deficiency as the cause of bleeding. MCV: Mean Corpuscular Volume; PT: Prothrombin Time; INR: International Normalized Ratio; aPTT: Activated Partial Thromboplastin Time; vWF: Von Willebrand factor; FVIII: Coagulation factor VIII; FXI: Factor XI

Parameter	Patient value	Reference range	Comment
Haemoglobin (g/dL)	9.4	12–16	Severe anaemia
Haematocrit (%)	29.5	36–46	Low
MCV (fL)	72	80–96	Microcytic
Platelets (×10⁹/L)	298	150–400	Normal
PT (s)	13.2	11–14	Normal
INR	1.02	0.8–1.2	Normal
aPTT (s)	45	26–35	Prolonged
vWF antigen (%)	94	50–150	Normal
vWF activity (ristocetin cofactor) (%)	86	50–150	Normal
FVIII activity (%)	72	50–150	Low-normal / transiently reduced
FXI activity (%)	0.3 (%); 1.24 (%) on repeat	70–150	Severely reduced

## Discussion

In women with inherited coagulation disorders, factor XI (FXI) deficiency represents an uncommon yet clinically significant cause of abnormal uterine bleeding (AUB). Among affected women, heavy menstrual bleeding (HMB) is the most frequent haemorrhagic manifestation, reported in up to 57-74% of cases, compared with approximately 29% in control populations [1,3,10]. This bleeding pattern is typically not only more abundant but also prolonged, substantially affecting quality of life.

FXI deficiency also increases the risk of perioperative bleeding during gynaecological and obstetric interventions, including hysterectomy [1,2]. A published case described severe postoperative haemorrhage following total abdominal hysterectomy in a woman with FXI activity between 2% and 5%, despite preoperative prophylaxis with fresh frozen plasma (FFP) [11]. This underscores the complexity of achieving haemostatic control in such patients, given the variable and often unpredictable relationship between plasma FXI levels and bleeding severity.

Similarly, patients with other coagulation disorders, such as von Willebrand disease, exhibit a higher incidence of perioperative bleeding and transfusion requirements. The predominance of fibrinolytic activity in genitourinary tissues further compounds the bleeding risk in FXI deficiency [1,2,3,12,13].

In contrast, the introduction of minimally invasive surgical techniques, such as total laparoscopic hysterectomy (TLH), has markedly improved outcomes in these high-risk patients. As reported in the present case and by Zheng et al., TLH is associated with lower intraoperative blood loss, reduced inflammatory response, and faster recovery compared with open approaches [7]. These advantages are particularly relevant in women with underlying coagulopathies, where limiting surgical trauma directly contributes to improved haemostatic stability.

The perioperative management of FXI deficiency remains challenging, as bleeding tendency correlates poorly with FXI activity. Management should therefore be individualised, based on the patient’s bleeding history, procedural risk, and comorbidities. Therapeutic strategies include antifibrinolytic agents (e.g., tranexamic acid), fresh frozen plasma (FFP), FXI concentrates, prothrombin complex concentrates, and recombinant activated factor VII (rFVIIa) where appropriate [3].

Although FXI concentrates provide targeted replacement and achieve predictable plasma levels, their availability is limited in many healthcare systems, and they carry a recognised thrombotic risk, particularly in older patients or those with cardiovascular risk factors. Prothrombin complex concentrates and rFVIIa may offer rapid haemostatic support but similarly increase thrombotic risk and are generally reserved for refractory cases. In contrast, tranexamic acid is widely accessible, inexpensive, and effective in reducing fibrinolysis but does not replace the missing factor [10].

FFP remains the most accessible option in many centres, particularly in low- and middle-resource settings. Its advantages include broad availability and the ability to provide FXI replacement when other products are not accessible. Limitations include the need for relatively large infusion volumes to achieve haemostatic levels and the associated risk of volume overload. In this case, FFP was selected because FXI concentrates and other factor-specific products were not available in our setting, and because the haematology team had extensive experience using FFP-based regimens combined with antifibrinolytic therapy. This approach provided adequate perioperative haemostatic control with favourable clinical outcomes [10,12,13].

Recent systematic reviews highlight that prophylactic use of these therapies significantly reduces the risk of postpartum and perioperative haemorrhage. Although FFP remains the most accessible replacement therapy in many healthcare settings, it carries the risk of volume overload when large transfusion volumes are required to achieve haemostatic levels. An emerging alternative is therapeutic plasma exchange (TPAP), which provides effective FXI replacement while maintaining euvolemia and minimising transfusion-related complications [13].

## Conclusions

Abnormal uterine bleeding in women with FXI deficiency represents a clinically relevant and frequently underestimated manifestation of this coagulopathy. The absence of a direct correlation between FXI plasma levels and bleeding severity necessitates a highly individualised, multidisciplinary approach.

Optimal outcomes depend on meticulous preoperative planning, early haematological involvement, and judicious use of prophylactic therapy. The combination of fresh frozen plasma or plasma exchange with antifibrinolytic agents offers an effective strategy to prevent surgical haemorrhage and improve recovery. Moreover, the adoption of minimally invasive procedures, such as total laparoscopic hysterectomy, further reduces intraoperative bleeding risk and enhances postoperative safety, ultimately improving quality of life in affected patients.
